# High-flow nasal cannula versus standard oxygen therapy assisting sedation during endoscopic retrograde cholangiopancreatography in high risk cases (OTHER): study protocol of a randomised multicentric trial

**DOI:** 10.1186/s13063-020-04378-z

**Published:** 2020-05-29

**Authors:** Venkatesan Thiruvenkatarajan, Ashok Dharmalingam, Gilberto Arenas, Medhat Wahba, Reinhard Steiner, Vasanth Rao Kadam, Andre Tran, John Currie, Roelof Van Wijk, Anthony Quail, Guy Ludbrook

**Affiliations:** 1 The Queen Elizabeth Hospital, Woodville, South Australia 5011 Australia; 2 Discipline Acute Care Medicine, The University of Adelaide, Adelaide, South Australia Australia; 3 Department of Anaesthesia, John Hunter Hospital, New Lambton Heights, New South Wales Australia; 4 The Royal Adelaide Hospital, Adelaide, South Australia Australia; 5 Flinders Medical Centre, Bedford Park, South Australia Australia; 6 Department of Anaesthesia, The Queen Elizabeth Hospital, Woodville, South Australia Australia; 7 Human Physiology Anaesthesia and Intensive Care, School of Medicine and Public Health, The University of Newcastle, Callaghan, New South Wales Australia

**Keywords:** Endoscopic retrograde cholangiopancreatography, Oxygen therapy, Low-flow oxygen cannula, High-flow oxygen cannula, Hypoxia

## Abstract

**Background:**

Endoscopic retrograde cholangiopancreatography (ERCP) is an increasingly common intervention in the treatment of pancreaticobiliary disorders. Patients are often elderly with complex co-morbidities. While monitored anaesthesia care with sedation is commonly used for most cases, few would require general anaesthesia with an endotracheal tube. Both low-flow and high-flow nasal cannulas (HFNC) are established ways of delivering supplemental oxygen, but it is unclear whether one technique is better than the other. HFNC seems a promising tool for advanced procedures but evidence to support its application in high-risk ERCP cases is limited. The rate of oxygen desaturation during endoscopy has been reported to be as high as 11%–50% and the method of oxygen delivery for ERCP merits further study.

**Methods/design:**

This is a prospective, randomised, multicentre trial comparing the efficacy of oxygen supplementation through HFNC versus low-flow nasal cannula during ERCP, in a cohort of patients at risk of adverse respiratory events. A total of 132 patients will be recruited across three sites and randomly assigned to either the low-flow or the HFNC group. The primary outcome is the proportion of patients experiencing hypoxia, defined by any event of SpO2 < 90%. The secondary outcomes include parameters centred on oxygenation, requirement of airway manoeuvres, successful completion of procedure, perioperative complications, patient satisfaction and cost analysis of the consumables. An intention-to-treat principle will be applied while analysing.

**Discussion:**

The demand for ERCPs is likely to increase in the future with the aging population. Our study results may lead to improved outcomes and reduce airway-related complications in patients undergoing ERCPs. The results will be presented at national and international meetings and published in peer-reviewed journals.

**Trial registration:**

www.ANZCTR.org.au, CTRN12619000397112. Registered on 12 March 2019.

## Background

Endoscopic retrograde cholangiopancreatography (ERCP) is a common intervention in the treatment of biliary and pancreatic diseases, and the demand for ERCP is increasing. There are several difficulties for the anaesthetist to deal with. It is generally performed in a prone or lateral position under moderate to deep sedation or general anaesthesia [1–3]. In most hospitals, ERCP is usually performed outside the operating room. The patients who require this procedure are typically elderly with significant co-morbidities. General anaesthesia with an endotracheal tube may be a ‘safe option’ in the prone position in terms of having a secured airway and a lower ERCP failure rate [3]; there may be a reduction in complication rates, but intubation has drawbacks. In addition to the well-known problems associated with insertion of the tube, managing a paralysed intubated patient prone creates its own challenges. Note that there is frequently a prolongation of anaesthetic time concurrent with the use of muscle relaxants.

Deep sedation using propofol is perhaps the most commonly employed technique for ERCPs. ERCP generally requires a deeper level of sedation compared to simple gastroscopy and there is an increased risk of both partial and complete airway obstruction. The goals of deep sedation are: preserving adequate spontaneous ventilation; maintaining cardio-respiratory stability; early recovery; and minimising any hypoxaemia at all times. Hypoxemia and aspiration have occurred when these procedures have been performed under deep sedation, especially in high-risk cases [4]. Reasons for desaturation during ERCP under deep sedation have been attributed to reduced cardiopulmonary reserve, advanced age, respiratory depression, duration of the procedure, and prone positioning. The incidence of hypoxemia during any endoscopic procedure is in the range of 11%–50% [5–7] and it is possibly as high as 60% with ERCP [8] (definitions of hypoxia vary between the studies). Prolonged hypoxia is a major risk factor for periprocedural cardiac arrhythmias and myocardial ischaemia [9–11]. Any methods of avoiding such occurrences is worthy of investigation.

Regardless of the level of sedation, supplemental oxygen is regularly administered to all patients to prevent hypoxia. Conventionally, it is delivered via nasal prongs and/or an extension tubing attached to the mouth guard (the application of the endoscope in the oral cavity precludes the use of normal face masks). The recommended flow rate through the low flow nasal cannula is 2–4 L/min, while the inspired FIO_2_ (fractional inspired oxygen concentration) is dependent on the ventilatory minute volume, in the range of 0.27–0.50 [12, 13]. Patients with high American Society of Anesthesiologists (ASA) status (III) [5, 14], high body mass index (BMI) [5, 15] and those with obstructive sleep apnoea (OSA) [16] are especially at an increased risk of hypoxia during advanced endoscopic procedures such as ERCP; these patients will obtain most benefit from any improvement in oxygenation techniques. It has been the case that superior oxygen delivery options preserving spontaneous respiration without interfering with the pharyngeal cavity have been limited for these procedures.

High-flow nasal cannula (HFNC) is a new approach for improving oxygenation and ventilation that has gained popularity in procedural sedation. It has been demonstrated to provide better oxygenation compared to the venturi face mask and low-flow nasal cannula during intravenous sedation for both bronchoscopy and dental procedures [17, 18]. HFNC can provide a maximum flow up to 70 L/min and delivers a flow-dependent positive airway pressure which increases end-expiratory lung volume and thereby improves oxygenation [19]. It has numerous physiological advantages that are not possible through standard (ventilation dependent) low-flow delivery systems. These advantages include the ability to create PEEP (up to 5–7.5 cm H_2_0), reduce the work of breathing, provide constant FIO_2_ up to 100%, provide good humidification, and aid washout of the pharyngeal dead space [19, 20]. In one centre, introducing the option to use HFNC affected anaesthetic practice and decreased the usage of general anaesthesia with an endotracheal tube (for a mixed group of ERCP and endobronchial ultrasound procedures) [21]. This observational study also showed HFNC also decreased the anaesthesia only time.

High-flow nasal oxygen has been described in the critical care setting for respiratory impairment for nearly a decade [20]. It has been successfully employed in various perioperative settings such as preoxygenation and airway management, including awake intubation [22, 23]. In our own institution, we have used HFNC for endoscopy and ERCP procedures in high-risk cases and have found it convenient to use and frequently beneficial. Being administered nasally, there is nil interference with the endoscopic insertion and manipulation in the oropharynx.

The aim of the OTHER (Oxygen Therapy in High risk ERCP) trial is to assess the efficacy and safety of oxygen supplementation achieved through HFNC compared with low-flow nasal cannula during ERCP in a cohort of patients at risk of adverse respiratory events. We hypothesise that the application of high-flow nasal oxygen will reduce the incidence of major respiratory adverse events and any resulting cardiovascular problems. Measurements will be made of respiratory and haemodynamic parameters, recovery profile and patients’ satisfaction.

## Methods/design

### Study design and setting

**Fig. 1:**
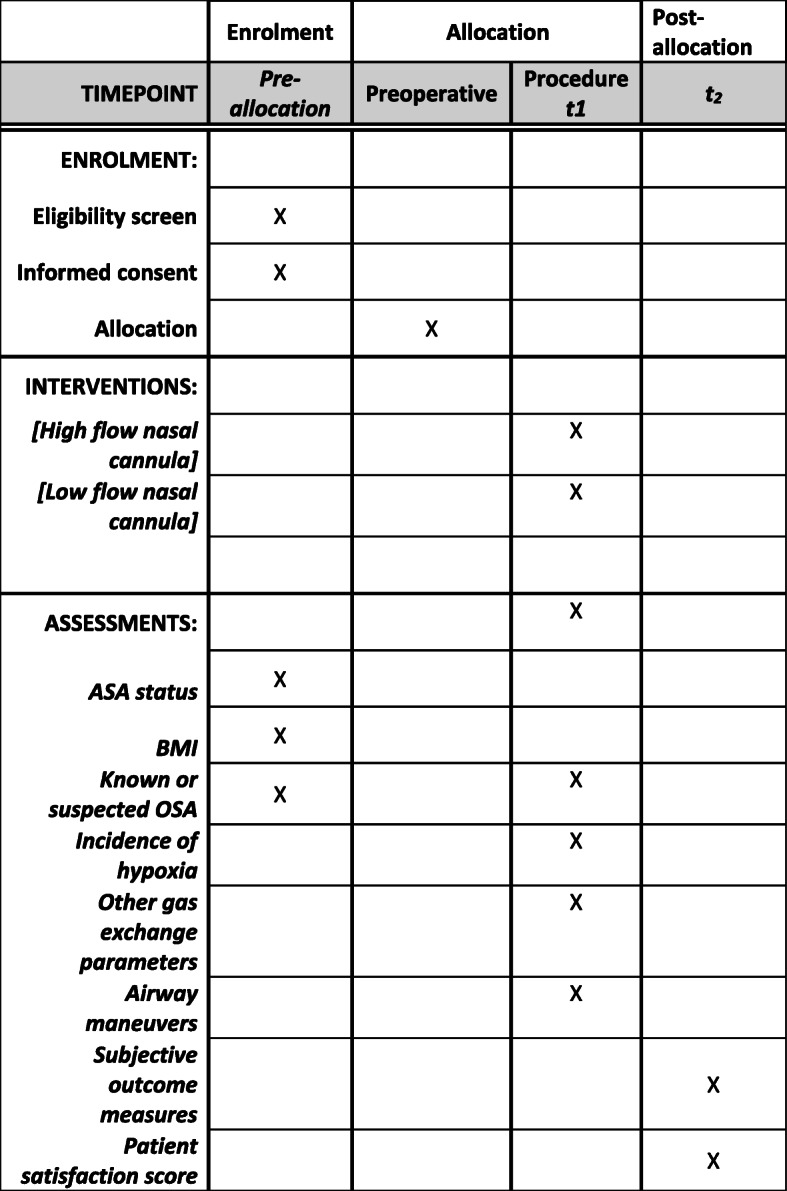
SPIRIT flow diagram: the schedule of enrolment, interventions and assessments. t1 is at the time of allocation and during the procedure, t2 is post anaesthesia care unit at the time of participants leaving the area and the study ends here. ASA American Society of Anaesthesiologists, BMI body mass index, OSA obstructive sleep apnoea

We will conduct this prospective multicentre randomised trial as per the recommendations for interventional trials (SPIRIT, Fig. 1 and Additional file 1) [24]. The final reporting of this trial will be in accordance with the Consolidated Standards of Reporting Trials (CONSORT) statement.

The OTHER trial will be conducted across three Australian hospitals: The Queen Elizabeth Hospital and Royal Adelaide Hospital, South Australia, and John Hunter Hospital, New South Wales. A total of 132 patients will be recruited. ERCP procedures are unique in terms of their presentation. A good proportion of them are undertaken as semi-urgent cases (unlike other pure electively planned cases) as inpatients and they may not get the opportunity to present to a pre-admission clinic. We are planning to recruit these patients at least 2 h before the procedure or whenever they are being assessed for anaesthesia. During this time, the patient information sheet will be provided. The recruitment and consenting are done by one of the investigators or by the anaesthetists assessing these patients. The pre-screening process would be based on the inclusion criteria mentioned below.

### Randomisation

**Fig. 2:**
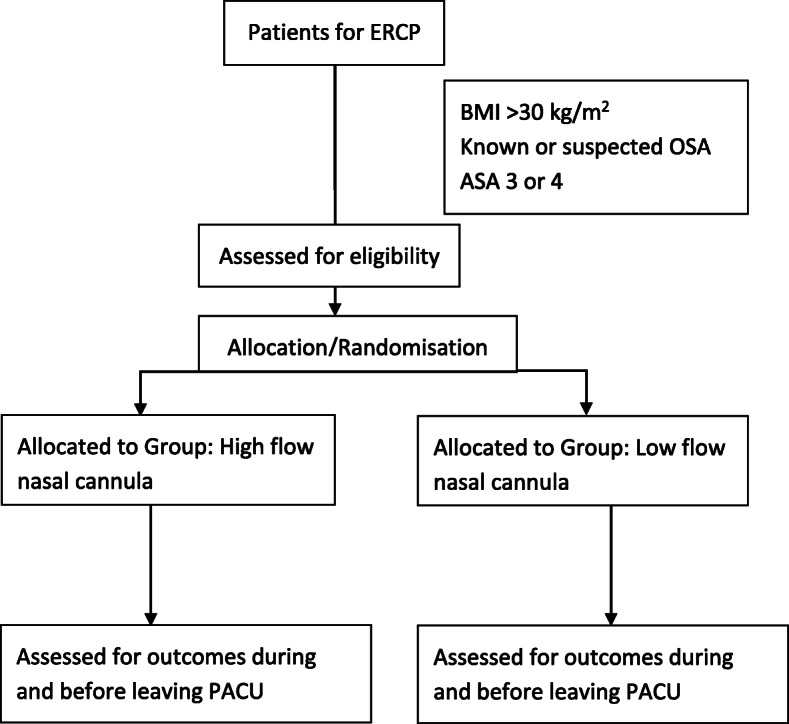
Study flow chart. ASA American Society of Anesthesiologists, BMI body mass index, ERCP endoscopic retrograde cholangiopancreatography, OSA obstructive sleep apnoea, PACU post-anaesthesia care unit

Participants will be randomly assigned to either the low-flow nasal oxygen or the HFNC group at a ratio of 1:1 (Fig. 2). The randomisation scheme will be generated by the Clinical Trials Division of the Pharmacy Department at The Queen Elizabeth Hospital. To ensure equal distribution of the intervention arm, stratification is done in specific blocks to predetermined numbers known only to the clinical trials division. This will be revealed only at the end of the trial. The random numbers and the group assignment will be supplied in sealed envelopes and handled only by the principal investigator. A set of 25 envelopes will be dispatched and used at each site, and an additional set of envelopes will be dispatched in blocks of 20 once the first 25 are used. The envelopes will be opened just before commencing ERCP and groups are allocated to the treatment intervention. Participants and the investigators are not blinded to the allocation. The patient information sheet contains descriptions of the trial. Diagrams of HFNC as well as low-flow nasal cannula are depicted in the sheet. Further, when we commence HFNC, we are obliged to explain to the patient that they will feel a high flow of oxygen coming through their nostrils. These are the reasons why we could not blind the participants to the intervention.

Data will be collected only by the investigators. The data analysts will be blinded to the intervention.

### Inclusion criteria

Adults (aged > 18 years) fulfilling any of these criteria: ASA 3 or 4; obesity (BMI > 30 kg/m^2^); obstructive sleep apnoea diagnosed either by polysomnography; being treated with CPAP for OSA; suspected OSA based on STOP BANG score >/3 (STOP BANG is an acronym for Snoring, Tiredness, Observed choking/gasping, blood Pressure elevation, BMI increase, Age, Neck size, male Gender).

### Exclusion criteria

Participants fulfilling any of the below criteria will be excluded: (1) deemed ‘difficult airway’ and/or difficult intubation based on clinical judgement and known previous difficult airway; (2) severe cardio-respiratory compromise or any other indications that necessitate the procedure to be done under general anaesthesia with endotracheal tube; (3) patients judged to be at significant risk of pulmonary aspiration. Risk assessment will be based on patient history (focusing particularly on risk factors for aspiration) and physical examination. Possible risk factors for aspiration include: increased gastric content; delayed gastric emptying; including lap band in situ; lack of fasting (< 6 h for solids and 2 h for clear fluid); increased regurgitation risk: uncontrolled or symptomatic gastro-oesophageal reflux, oesophageal strictures, Zenker diverticulum and achalasia; laryngeal incompetence due to cerebral infarct, head injuries, neuromuscular disorders (Parkinson’s disease, Gullian Barre), muscular dystrophies (cerebral palsy, cranial neuropathies); and (4) emergency surgery and any other criteria warranting general anaesthesia with an endotracheal tube.

### Intervention and comparison

Patients will be randomly allocated (computer-generated randomisation) to either the low-flow nasal oxygen group (group L; n = 66) or the high-flow nasal oxygen group (group H; n = 66). In group L, the procedure will be performed under deep sedation and analgesia, with supplemental oxygen 4 L/min via regular nasal cannula and a further supplemental oxygen source with a flow rate of 4 L/min administered through an extension tubing attached to the mouth guard. In group H, the procedure will be performed under deep sedation and analgesia similar to Group L, with oxygen delivered through a HFNC. This will be accomplished using the Optiflow THRIVE (Transnasal Humidified Rapid Insufflation Ventilatory Exchange) device (Opti-Flow, Auckland, New Zealand). Flow rate through the cannula will be commenced at 30 L/min and fractional inspired oxygen concentration will be set at 100%. The flow will be gradually increased after the administration of sedative agents and will be maintained at 50 L/min during the procedure. The flow rate may be either increased up to 70 L/min if necessary or decreased to 30 L/min if the patient does not tolerate the higher flow rate (50 L/min is very well tolerated in even very lightly sedated patients).

Standard monitoring will be applied, including continuous electrocardiogram, blood pressure automatically measured every 3 min, transcutaneous capnography and pulse oximetry. The procedures will be done in either the lateral or prone positions. A standard sedation technique will be applied for both groups. Sedation is to be provided by titrated doses of fentanyl 0.5–1.0 mcg/kg, as required, and a propofol target controlled infusion (using the Marsh model) commencing at an initial plasma target of 1.5–2.0 mcg/mL and titrated up or down during the procedure, between target values of 1–4 mcg/mL based on satisfactory depth of anaesthesia and frequency of ventilation (8–14 breaths per min). Administration of further fentanyl top up doses (25 mcg) will be at the anaesthetist’s discretion. In all cases, airway obstruction needs to be avoided.

If it is deemed necessary that during the intervention phase, if the participants’ clinical condition warrants an advanced airway such as tracheal intubation, it will be undertaken and they will be retained in an intention-to treat analysis.

### Outcome measures

#### Primary outcome

The occurrence of hypoxia, defined by any event of SpO2 (oxygen saturation measured by pulse oximetry) < 90% of any duration, will be compared between the two groups.

#### Secondary outcomes

Secondary outcomes are as follows:

- Number of events of hypoxia, defined as desaturation < 90%. The mean number of events during the procedure will be compared between the two groups;
- Lowest recorded SpO_2_ during the procedure;
- Transcutaneous CO_2_. Maximum value recorded and average value during the case. The mean values during the procedure will be compared between the two groups;
- Lowest recorded SpO_2_% during the procedure. The mean values during the procedure will be compared between the two groups;
- Requirement of minor airway manoeuvres: jaw lift/jaw thrust, nasopharyngeal airway insertion. Proportion of patients requiring these manoeuvres will be compared between the two groups;
- Requirement of major airway manoeuvres: bag mask ventilation, endotracheal intubation. Proportion of patients requiring these manoeuvres will be compared between the two groups;
- Arrhythmia (any change in cardiac rhythm observed on the electrocardiogram). Proportion of patients manifesting arrhythmia during the procedure will be compared between the two groups;
- Total fentanyl dose. The mean values (the doses in micrograms) during the procedure will be compared between the two groups;
- Requirement of antispasmodic agent. Proportion of patients requiring this medication will be compared between the two groups;
- Total duration of procedure (starting from sedation until leaving the suite). The mean duration in minutes will be compared between the two groups;
- Duration under sedation/anaesthesia: measured from the time of induction till eye opening. The mean duration in minutes will be compared between the two groups;
- Successful completion of the procedure: Yes/No. Proportion of patients fulfilling this criterion will be compared between the two groups;
- Early complications – elicited as patients leave recovery;
- Dry mouth/nose/throat: binary outcome (constant pain or discomfort in the mouth/nose/throat). Proportion of patients experiencing this adverse event will be compared between two groups:
  - ◦ Sensation of abdominal bloating: Y/N. Proportion of patients experiencing this adverse event will be compared between the two groups;
- Patients’ satisfaction score on leaving recovery: 5 points numerical rating scale:
  - ◦ Very satisfied (5), somewhat satisfied (4), neither satisfied nor dissatisfied (3), somewhat dissatisfied (2), very dissatisfied (1). Proportion of patients at a particular threshold will be compared between the two groups;
- Cost analysis of the consumables: low flow versus high flow. This will be analysed for only the consumables required during the procedure for an average case and compared across the two groups. The information will be obtained from our clinical pharmacy and anaesthetic nursing department.

### Sample size calculation

A 21.4% incidence of hypoxia (SPO_2_ < 90% for 15 s) was noted in an Australian study during ERCP with propofol sedation in older patients (20). Assuming a 16% reduction in hypoxic events by employing the HFNC technique (given the rate of hypoxia as 21.4% in the sedation group and an estimated 5.4% in the HFNC group), with a power of 80% and an alpha error of 0.05, a total of 132 patients would be required (66 in each arm).

### Statistical analysis

Mean and standard deviation values will be estimated for continuous outcomes while frequency and percentage will be computed for binary outcomes. 95% confidence intervals around the point estimate will be calculated where appropriate for the primary and secondary outcomes. Descriptive statistics will be used to present the results. *P* < 0.05 will be considered significant. Analyses will be intention-to-treat from randomisation. All randomised cases will be included in the analyses, regardless of missing data. As the data capture is only limited to a few hours after the intervention and the investigators are directly involved in the conduct of the study, we anticipate very few missing data. A subgroup analyses will be attempted (if feasible) for the high BMI and OSA groups combined.

### Strengths and limitations of this study

Our study is the first multicentre randomised controlled trial comparing low-flow versus high-flow nasal oxygen therapy for improving oxygenation in high-risk patients for ERCP. There are standardised and objective endpoints. In addition, patient-reported outcome measures are explored. The application of transcutaneous CO_2_ measurements helps to overcome the limitation of expired CO_2_ monitoring during ERCP. The nature of the study precludes blinding of the participants and the anaesthetists, which may contribute to bias and influence the results.

### Quality control and data monitoring

The investigators and anaesthetic nursing staff will undergo special training on using and troubleshooting the transcutaneous CO_2_ equipment. Only data on respiratory, haemodynamic and perioperative outcome endpoints will be collected during the procedure and it is not time bound. Only one set of data will be collected in the post-anaesthesia care unit when they leave the area. The data will be de-identified when entered into an Excel spreadsheet for analysis. No blood or tissue samples will be collected. The original data collection will be kept for cross-checking. The data forms will be securely placed in our department’s locked filing cabinets. Any serious adverse events across any sites will be notified within 24 h to the principal investigator. Patient recruitment and data quality will be regularly checked by the investigators across the trial centres. The study involves comparing established standard practices and hence deemed as low risk to be specifically monitored by a data monitoring committee.

The electronic data will be stored in the department’s computers with password protection. After the analysis, only the principal investigator will have access to the data. A strict privacy policy will be maintained by all investigators to protect confidentiality throughout the trial process. This study will be performed in accordance with the Standard Protocol Items: Recommendations for Interventional Trials [SPIRIT] recommendations.

### Ethics and dissemination

This study has been approved by the Central Adelaide Local Health Network Human Research Ethics Committee (Version 4, dated 31 October 2018; approval date 25 September 2018; ethics approval no. Q20180807) as well as by the Hunter New England Local Health District Research governance (NSW REGIS no. 2019PID00554, SSA reference no. 2019/STE0047). This study was registered 1 month after the first patient was recruited at www.ANZCTR.org.au (CTRN12619000397112) on 12 March 2019 and registered at Australian New Zealand Clinical Trials Registry (ANZCTR) (registration no. ACTRN12619000397112). The results of the study will be disseminated through peer-reviewed publications and national/international conference presentations. Protocol amendments will be immediately notified to all centres.

## Discussion

The increasing demand for ERCPs on a cohort of patients with multiple co-morbidities, along with time constraints in optimising these patients when it is attempted on a semi-urgent basis, has generated an enormous interest in the quest for exploring better airway management strategies. HFNC is one such device that has been attempted across a variety of settings requiring sedation. Besides the primary outcome assessing the occurrence of hypoxia, secondary outcomes of airway intervention and patient-reported scales are also measured.

The risks are very negligible from these two nasal cannulas. They have been in use for many years as standard methods of delivering oxygen. The patients will be asked for symptoms of dryness of mouth or throat or nose after the procedure as part of outcome measures. If dryness develops, it normally settles within a few hours. These outcome measures are aimed at detecting some of the known side effects of oxygen delivery. The HFNC can cause abdominal distension, particularly in children after prolonged use. At the end of this procedure, endoscopists routinely evacuate any insufflated gas from the stomach and this will mitigate any such risk in this group. There are no other specific safety issues concerning HFNC for participants and administering staff.

The results may lead to improved outcomes and reduce airway-related complications in patients undergoing ERCPs. In our opinion, the results will also deliver meaningful information on the role of HFNC-assisted sedation in any setting.

## Trial status

The recruitment commenced in February 2019 and the trial is expected to be completed by September 2020. Currently, the trial is on hold in view of the aerosolisation concerns with HFNC therapy during the COVID-19 outbreak.

## Supplementary information

**Additional file 1.** SPIRIT Checklist.

## Data Availability

As no datasets were generated or analysed for this trial at this stage, data sharing is not applicable. The Principal Investigator VT will have access to the final trial dataset. Trial and data details can be obtained from the principal investigator upon reasonable request.

## Abbreviations

ERCP: Endoscopic retrograde cholangiopancreatography
HFNC: High-flow nasal cannula
FIO_2_: Fractional inspired oxygen concentration
ASA: American Society of Anesthesiologists
BMI: Body mass index
OSA: Obstructive sleep apnoea
OTHER: Oxygen Therapy in High risk ERCP
SpO2: Oxygen saturation measured by pulse oximetry
CO_2_: Carbon dioxide
THRIVE: Transnasal Humidified Rapid Insufflation Ventilatory Exchange
